# Effect of broccoli extract supplement on carcass traits and lipid metabolism in Holstein steers

**DOI:** 10.3389/fgene.2025.1618682

**Published:** 2025-07-25

**Authors:** Shuangshuang Chen, Xinyu Zhang, Tao Zhu, Die Tang, Mingxing Wen, Chuang Tang, Ling Hou, Zhiyao Zeng, Shanfeng Tong, Xuelong Li, Lu Lu, Keren Long, Quanhui Peng, Anan Jiang, Jideng Ma

**Affiliations:** ^1^State Key Laboratory of Swine and Poultry Breeding Industry, College of Animal Science and Technology, Sichuan Agricultural University, Chengdu, China; ^2^ Chengdu Xunchi Youniu Agricultural Technology Co., Ltd., Chengdu, Sichuan, China; ^3^ Ningxia Bahe Animal Husbandry Co., Ltd., Haiyuan County, Zhongwei, Ningxia, China; ^4^Institute of Animal Nutrition, Sichuan Agricultural University, Chengdu, Sichuan, China

**Keywords:** Holstein steer, growth and fattening, broccoli extract, lipid metabolism, 16S, metabolomics, transcriptomics

## Abstract

**Introduction:**

Feed additives are widely used to enhance feed efficiency and promote animal growth and health. Broccoli extract, a plant-derived additive rich in bioactive compounds, has potential physiological regulatory effects. However, its specific impact on cattle remains unclear.

**Methods:**

This study investigated the effects of broccoli extract supplementation on growth performance, rumen microbial composition, blood metabolites, and gene expression in the liver and adipose tissue of castrated Holstein bulls. Animals were randomly assigned to three groups and supplemented daily with 0 g, 15 g, or 18 g of broccoli extract for 45 days.

**Results:**

No significant differences were observed among groups in average daily gain, dressing percentage, or fecal score (P > 0.05). However, broccoli extract supplementation significantly improved feed intake, lying time, rumination rate, and net meat yield, while reducing subcutaneous fat percentage (P < 0.05). 16S rRNA sequencing revealed increased rumen microbial diversity in the 18 g group. Blood metabolomics showed elevated prostaglandin E2 levels and enrichment in pathways related to inflammation and lipid metabolism. Transcriptomic analysis revealed enrichment of pathways associated with immune responses and lipid regulation. Integrated multi-omics analysis further demonstrated strong correlations between lipid-related metabolites and gene expression patterns.

**Conclusion:**

Broccoli extract supplementation modulated feeding behavior and rumen microbiota, improved carcass traits, and influenced lipid metabolism and inflammation-related pathways in Holstein cattle. These findings highlight its potential as a functional feed additive for improving beef cattle production.

## 1 Introduction

In beef cattle production, effectively controlling fat deposition while ensuring weight gain remains a critical challenge for achieving efficient production and improving meat quality. In fattening cattle, fat is primarily distributed across subcutaneous, intermuscular, and visceral depots, with certain fat deposits—such as visceral fat—considered economically unproductive carcass components. These not only reduce lean meat yield and carcass utilization but also adversely affect meat quality and may contribute to the development of various metabolic disorders (Melendez et al., 2019). It is well established that the core objective of premium beef production is to increase lean meat yield and the proportion of marketable meat while controlling production costs. Therefore, precise regulation of fat deposition processes is essential. Fat deposits were observed in castrated bulls compared to uncastrated bulls (Mach et al., 2009; Zhou et al., 2011; Purchas et al., 2002). The main sites where fat deposition usually occurs are the liver and adipose tissue, where the liver is the central organ for the uptake, oxidation, and metabolic transformation of nonesterified fatty acids (Nguyen et al., 2008; Nafikov and Beitz, 2007; Ramayo-Caldas et al., 2012). Adipose tissue is the main fat depot, especially in the subcutaneous and visceral regions. When the body needs energy, fat cells will break down triglycerides into free fatty acids for other tissues to use (Ghaben and Scherer, 2019). Plant-derived extracts are biologically active substances with unique functional groups that are sustainable additives that can be used to enhance animal productivity and health (Lipiński et al., 2017). Such extracts have antioxidant, antimicrobial, anti-inflammatory, and immune-stimulating properties (Gessner et al., 2017), and some of them have a beneficial regulatory effect on the lipid metabolism of ruminant animals (Morán et al., 2012; Inserra et al., 2014). Additionally, adding plant-derived extracts to feed has been shown to alter the microbial composition and activity in the rumen and inhibit the growth of harmful bacteria, thus regulating rumen fermentation in ruminant animals (Lin et al., 2012; Klevenhusen et al., 2012; Jafari et al., 2016; Cappucci et al., 2018; Cardozo et al., 2005). Because plant-derived extracts have diverse chemical structures, animals react and interact with them in different ways (Perron and Brumaghim, 2009).

Broccoli (*Brassica oleracea* L. italica, Cruciferaceae) is a candidate functional food with antioxidant properties that may help reduce the risk of some cancers (Latté et al., 2011; Steinkellner et al., 2001; Paturi et al., 2010), but little is known about lipid metabolism. Broccoli contains abundant glucosinolates, including Glucoraphanin. Glucoraphanin is converted to sulforaphane by myrosinase during the chopping and chewing of broccoli or in the digestive system of mammals (Ranaweera et al., 2022). Sulforaphane in broccoli extract can potentially have anti-obesity effects by normalizing the expression of lipid metabolism-related genes (Ranaweera et al., 2022). In addition, sulforaphane can induce adipocyte Browning and promote glucose and lipid utilization (Zhang et al., 2016). Broccoli fiber and high-fiber corn oil can reduce serum cholesterol and triglycerides, and alter the expression of genes involved in lipid synthesis in the liver, which may have beneficial effects on lipid metabolism (Nyanhanda et al., 2012). Paula Aranaz and colleagues (Aranaz et al., 2019) investigated the effects of supplementing broccoli extract on rat body weight gain, food efficiency, obesity accumulation, cholesterol metabolism, liver fat deposition, and potential regulation of fat cell metabolism.

**FIGURE 1:**
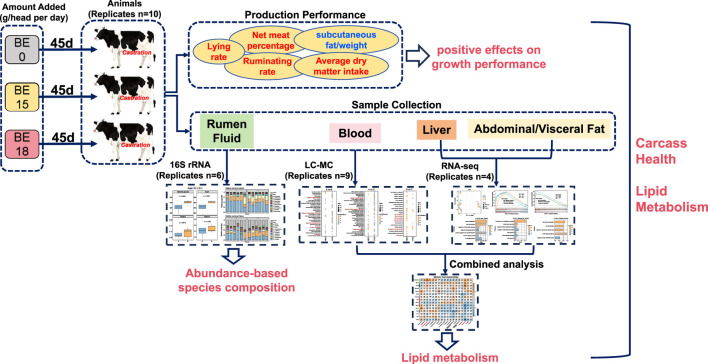
Overview of the integrated multi-omics workflow exploring the impact of broccoli extract on lipid metabolism in Holstein steers. Three experimental groups were established, with each steer receiving daily doses of broccoli extract (0g, 15g, 18g). In terms of production performance, broccoli extract significantly improved animal behavior and carcass traits, including increased rumination rate, lying time, feed intake, and net meat yield, while significantly reducing subcutaneous fat proportion. Multi-omics analyses included 16S rRNA sequencing of rumen fluid, untargeted metabolomics of blood, and transcriptomics of liver and abdominal/visceral fat. The combined analysis of metabolomics and transcriptomics revealed the synergistic changes between differential metabolites and genes. These findings highlight the comprehensive effects of broccoli extract on rumen health, blood metabolism, and lipid metabolism in Holstein steers, providing valuable scientific insights for its potential application in livestock production.

The addition of appropriate feed additives is known to significantly improve feedlot cattle productivity and promote feed intake and feed digestibility (Silvestre et al., 2023; Author anonymous, 2021; de Sá Assis et al., 2023). Here, as a natural plant extract that can promote lipid metabolism in the body, the effect of broccoli extract on the health of the cattle carcass has not been fully studied and thus aroused our great interest. Therefore, this study aimed to investigate the effects of broccoli extract additive on growth performance, ruminal microbial community characteristics, adipose and liver tissue gene expression, and blood metabolism in Holstein steers to explore its potential to improve cattle productivity and health (Figure 1).

## 2 Materials and methods

### 2.1 Animals and experimental design

The experimental design and animal grouping are shown in Table 1. Cattle were randomly assigned after uniform selection based on body weight and age. The broccoli extract was purchased from Fu Feng Ci Yuan Biotechnology Co., Ltd. The dosage selection was based on previously reported effective ranges of broccoli extract in animal models (such as rats), and further evaluated for feasibility and cost-effectiveness in the context of livestock production.

**TABLE 1:** Experimental design.

Experimental Group	Treatment	Number of animals	Weight (kg)	Broccoli extract dosage (g/day/head)	Duration of feeding (Days)
A	Control	10	700 ± 20	0	45
B	treatment	10	700 ± 20	18	45
C	treatment	10	700 ± 20	15	45

Data Recording: Feed intake, lying time rate, rumination rate, and weight data were recorded at the beginning and end of experiments for Groups A, B, and C. Carcass-related data were collected post-slaughter. Feed intake was calculated as total feed intake per group divided by the number of cattle multiplied by the number of days of the experiment. Lying time rate was calculated as the number of cattle lying down at noon divided by the total number of cattle × 100%. Rumination rate was calculated as the number of cattle ruminating at noon divided by the number of cattle lying down × 100%. Lying time rate and rumination rate data were collected 2–3 h after noon feeding. Dressing percentage was calculated as carcass weight divided by pre-slaughter live weight × 100%, and net meat percentage was calculated as net meat weight divided by pre-slaughter live weight × 100%.

Castration was uniformly performed at around 3 months of age, before the animals were assigned to experimental groups. The castration procedure was as follows. The method of holding in the supine position with the limbs raised and the left side horizontal holding were used. Routine shearing and disinfection. The testis was fixed with one hand, and the knife was held with the other hand to cut along the longitudinal axis of the testis. The incision was about three-quarters of the testis and was cut neatly. The testis is extruded and the common sheath is detached and pushed to the top. The other testicle was removed in the same way, the stump was smeared with a small amount of iodine tincture, the wound was sprinkled with an appropriate amount of Xiaoyan powder, and the wound was disinfected with iodine tincture.

### 2.2 Feeding and management

The experiment was conducted at the scale breeding farm of Gansu Xunchi Youniu Agriculture and Animal Husbandry Co., Ltd. from 28 August to 12 October 2023, in a region with a typical temperate continental arid climate.

The experimental cattle were raised in a loose housing system without being confined to individual pens. They were fed three times a day, with the feeding amounts distributed as follows: 25% in the morning, 40% at noon, and 35% in the afternoon. Free access to water was provided. If any leftovers were found after each feeding, the intake calculation was adjusted by subtracting the leftover amount from the total feeding amount.

How the broccoli additive is used: Mix the product with the appropriate amount of water to form a homogeneous solution. This solution was then slowly added to the preprepared feed, ensuring uniform coverage of each portion of feed. At the same time, the feed was gently stirred using a dedicated stirring device to ensure that the broccoli extract was well mixed and to avoid precipitation. Finally, the mixed feed was dispensed to ensure that it remained fresh during storage and feeding so that the cattle received only the best nutritional results. The alleyways of the cattle pens were cleaned daily, and the feces inside the pens were regularly cleaned and disinfected until sample collection 4 days later. The nutritional composition and levels were determined according to the recommended values of nutrition standards that correspond to the transitional period, pre-fattening period, and fattening period. Detailed composition and nutritional levels are given in Table 2.

**TABLE 2:** Composition and nutrient levels of basal diet.

Composition	Content (%)	Item	Nutrient levels
Corn silage	23.73	ME (Mcal/kg)	3
Corn straw	22	CP (g/kg)	146
Beer lees	8	NDF (g/kg)	219.7
Distill grains	20	ADF (g/kg)	117.3
Corn	12	Fat (g/kg)	44.6
Flaked maize	8	Ash (g/kg)	64.6
Soybean meal	3.88	Ca (g/kg)	7.9
CaHPO4	0.68	P (g/kg)	4.3
CaCO_3_	0.66		
NaCl	0.3		
NaHCO3	0.15		
Premix	0.6		
Total	100		

### 2.3 Sample collection

Samples were collected at a large-scale beef cattle farm in Gansu Province, China on 15 October 2022.

Blood collection: At the end of the experimental period, nine beef cattle were randomly selected from each of the three groups for blood collection and ear tags of the selected animals were recorded. Venous blood was drawn from the jugular vein of selected cattle using a disposable blood sampling needle and a 10 mL red vacuum collection vessel. Three tubes of blood were taken from each animal and made sure that each tube contained 6–7 mL of blood. After the whole blood sample was allowed to stand for 1 h, the serum was separated by centrifugation at 3,000 rpm for 15 min. Finally, the serum was transferred to a labeled 1.5 mL centrifuge tube and stored at − 80°C.

Tissue sample collection: Four beef cattle were randomly selected from group A and group b, respectively, and tissue samples were collected. The night before slaughter, fasting was performed. The selected cattle were weighed in the fasted and restrained state at 3a.m. the following morning. Slaughter was carried out by exsanguinating the jugular vein. Abdominal fat and visceral fat were isolated after dissection. When liver tissue was collected, surface dirt was adsorbed with filter paper, and the surface film was removed to obtain the middle part of liver tissue. Collected abdominal fat, visceral adipose tissue samples, and liver tissue samples were promptly stored in liquid nitrogen tanks for transcriptome sequencing.

Rumen fluid collection: Before morning feeding on the last day of the experiment, six beef cattle were randomly selected from group A and group B, respectively, and rumen fluid was collected using an oral collector. The first 50 mL of rumen fluid was discarded. Then 200 mL of rumen fluid was extracted, filtered through 4 layers of gauze, and transferred to 5 mL cryotubes. Samples were frozen in an 80°C refrigerator and used to determine the rumen microbiota.

### 2.4 Transcriptome sequencing methods and data analysis

Total RNA was isolated from the tissue samples using the HiPure Total RNA Mini Kit (Magen, Guangzhou, China) according to the manufacturer’s instructions. The integrity and quality of total RNA samples were analyzed with NanoDrop 2000 (Thermo Fisher Scientific, Wilmington, DE, United States) and Bioanalyzer 2,100 system (Agilent Technologies, Palo Alto, CA, United States). The RNAs with a ratio of absorbance at 260/280 nm ranging from 1.8 to 2.0 and RIN value >1.8 were selected for further study. Qualified samples were constructed for Library in Novogene (Beijing, China), and high-throughput sequencing was performed on the DNBSEQ-T7 platform. The low-quality reads were removed, namely, those with ≥10% unidentified nucleotides, >10 nt aligned to the adapter, and with >50% of bases with Phred quality <5.

Clean data were mapped to the bovine reference genome *Bos taurus* (ARS-UCD1.2) using STAR (v.2.6.0c). Transcript abundance for protein-coding genes (PCGs) was quantified as transcripts per million (TPM) using Kallisto (v.0.44.0), with default parameters. We defined expressed PCGs as those with TPM >1 in at least three samples, considering the expression characteristics of different transcripts.

Differential gene expression analysis was performed using the EdgeR (v 4.6.2) software package, where genes with |fold change| > 1 and FDR <0.01 were considered DEGs. (GO) terms and Kyoto Encyclopedia of Genes and Genomes (KEGG) pathways, was performed using the Metascape platform (v3.5.20250701; http://metascape.org). The analysis was conducted with a Qvalue cutoff of 0.05 and a minimum enrichment score of 1.5, based on the platform’s default settings. Gene set enrichment analysis (GSEA) was carried out using the R packages clusterProfiler (v 4.17.0), enrichplot (v 1.28.2), and DOSE (v 4.2.0). Weighted gene co-expression network analysis (WGCNA) was conducted using the WGCNA package (v1.72-1). Correlation analysis between differential metabolites and DEGs was performed using two complementary approaches. First, pairwise Pearson correlation analysis was applied to assess direct associations between individual DEGs and differential metabolites. Gene–metabolite pairs with |r| > 0.7 and P < 0.05 were considered statistically significant. Second, gene co-expression modules were identified using WGCNA with a soft-thresholding power of 9, and the resulting module eigengenes were correlated with metabolite profiles to identify module-level associations. Key gene–metabolite interaction networks were visualized using Cytoscape (v3.9.1).

### 2.5 Metabolomics mass spectrometry and data analysis

The prepared samples were subjected to extraction and separation on an ultra-high performance liquid chromatography (UHPLC) system equipped with a HILIC column. The chromatographic conditions were as follows: Column: Hypesil Gold (C18); Flow rate: 0.2 mL/min; Column temperature: 40°C. For positive ion mode, mobile phase A consisted of 0.1% formic acid, and mobile phase B was methanol. For negative ion mode, mobile phase A consisted of 5 mM ammonium acetate at pH 9.0, and mobile phase B was methanol. The initial three QC samples were used to monitor the instrument status and equilibrate the LC-MS system before injection. The following three QC samples were employed for segmented scanning, and the secondary spectra obtained from both QC and experimental samples were used for metabolite identification. QC samples were intermittently inserted throughout the sample analysis to assess system stability and ensure quality control throughout the experiment.

The scan range was set to m/z 100–1,500. The settings for the ESI source were as follows: Sheath gas flow rate: 35 psi; Spray voltage: 3.5 kV; Capillary temperature: 320°C; S-lens RF level: 60; Auxiliary gas flow rate: 10 L/min; Auxiliary gas heater temperature: 350°C; Polarity: positive and negative modes. MS/MS secondary scanning was performed using data-dependent scans.

The raw data files were imported into the CD v3.1 software for processing. For each metabolite, simple screening was performed based on parameters such as mass-to-charge ratio and retention time. Peak alignment was conducted for samples with retention time deviations of 0.2 min and mass deviations of 5 ppm to improve the accuracy of the identification. Then, the mass deviation was set to 5 ppm, signal intensity deviation to 30%, signal-to-noise ratio to 3, and minimum signal intensity and summed ion information were used for peak extraction. Peak areas were quantified, and target ions were integrated. Molecular formulas were predicted based on molecular ions and fragment ions, and compared with the mzCloud (https://www.mzcloud.org/), mzVault, and Masslist databases. Background ions were removed using blank samples, and the original quantitative results were standardized to identify and obtain the relative quantification of metabolites.

The identified metabolites were annotated using the KEGG (https://www.genome.jp/kegg/pathway.html), LIPIDMaps (http://www.lipidmaps.org/), and HMDB (https://hmdb.ca/metabolites) databases. For multivariate statistical analysis, the metabolomic data were transformed using the metaX software (v1.2.0), and principal component analysis (PCA) and partial least squares discriminant analysis (PLS-DA) were performed to obtain the Variable Importance in Projection (VIP) values for each metabolite. For univariate analysis, t-tests were performed to calculate the statistical significance (P-value) of each metabolite between two groups, and the fold change (FC) of metabolites between two groups was also calculated. The default criteria for differential metabolite selection were typically VIP >1, P-value <0.05, and FC ≥ 2 or FC ≤ 0.5. For pathway enrichment, identified metabolites were mapped to KEGG pathways, and statistical significance was evaluated with adjusted P-values (FDR <0.05) using MetaboAnalyst v5.0. Enrichment results were visualized accordingly.

### 2.6 16S rRNA sequencing and data analysis

DNA extraction was conducted using the QIAamp DNA stool kit (Qiagen, Hilden, Germany) following the instructions of the manufacturer with slight modifications. Briefly, 100 mg of lyophilized rumen was weighed and lysed by incubation for 5 min at 95°C. DNA elution was done with 100 μL of Buffer AE, after incubation for 10 min at room temperature. DNA quality was assessed by agarose gel electrophoresis and DNA concentration was determined using a NanoDrop ND-1000 spectrophotometer (NanoDrop Technologies, Wilmington, DE, United States).

Typically, target sequences that reflect bacterial composition and diversity, such as bacterial ribosomal RNA and specific gene fragments, are used as markers. Primers are designed based on their conserved domains, and sample-specific barcode sequences are added to enable PCR amplification of the variable regions of the rRNA gene (single or multiple contiguous regions) or specific gene fragments. During the PCR amplification process, we utilized high-quality DNA polymerase (Pfu) produced by FullGen Company, and strict control was maintained over the amplification cycles to ensure that each cycle was minimized. Additionally, a negative control must be established during this process to detect microbial contamination from the environment or reagents. If a negative control is obtained, it indicates contamination, and the subsequent experiments should not proceed.

The sequencing library was prepared using the TruSeq Nano DNA LT Library Prep Kit from Illumina. First, the amplified products were subjected to end repair, using the End Repair Mix 2 from the kit to remove overhanging bases at the 5′ end of the DNA sequence and to add phosphate groups to the 3′ end, filling in any gaps at the 3′ end of the DNA sequence. A base (A) was then added to the 3′ end of the DNA sequence to prevent self-ligation of the DNA fragments and to ensure that the target sequence could connect to the sequencing adapters. An indexing adapter containing a library-specific tag was added to the 5′ end of the sequence, allowing the DNA molecule to be anchored to the Flow Cell. Using BECKMAN AMPure XP Beads, magnetic beads were utilized to remove unligated fragments and purify the library system after adapter addition. The DNA fragments with the ligated adapters were then amplified via PCR to enrich the sequencing library templates, and the enriched library product was purified again using BECKMAN AMPure XP Beads. Finally, a 2% agarose gel electrophoresis was performed for the last round of selection and purification.

16S rRNA gene sequencing data were processed using QIIME 2 (v2024.10). Raw reads were quality filtered and denoised using the DADA2 plugin, which infers exact amplicon sequence variants (ASVs) without clustering. Taxonomic classification was assigned based on the SILVA database (release 138). Alpha diversity metrics, including observed ASVs, Shannon diversity index, and Chao1 richness estimator, were calculated to assess within-sample microbial diversity. Sequencing depth and coverage were evaluated by generating rarefaction curves, with a sampling depth set to the minimum number of reads among all samples. Beta diversity was estimated using Bray-Curtis dissimilarity and weighted and unweighted UniFrac distances based on ASV abundance tables. Statistical significance of beta diversity differences was tested using Permutational Multivariate Analysis of Variance (PERMANOVA) with 999 permutations. Differentially abundant taxa and potential biomarkers were identified using LEfSe (v1.1.0) with a Linear Discriminant Analysis (LDA) score cutoff of 2.0. Functional profiling of microbial communities was performed using PICRUSt2 (v2.6.2) with default parameters, based on KEGG Orthology (KO) pathway annotations.

### 2.7 Statistical analysis

SPSS 23.0 was used for statistical analysis, and Graphpad Prism 9.0 was used for graphic visualization. The normally distributed data were expressed as mean ± SD. Continuous data with normal distribution were analyzed using ANOVA. Non-normal distribution data were taken as logarithm and analyzed by ANOVA. Comparison between groups was performed by the least significant difference method (Least Significant Difference, LSD). *P < 0.05* was considered to be statistically significant (ns: *P* ≥ 0.05, *: *P* < 0.05, **: *P* < 0.01, ***: *P* < 0.001, ****: *P* < 0.0001).

## 3 Results

### 3.1 Impact on growth performance

**FIGURE 2:**
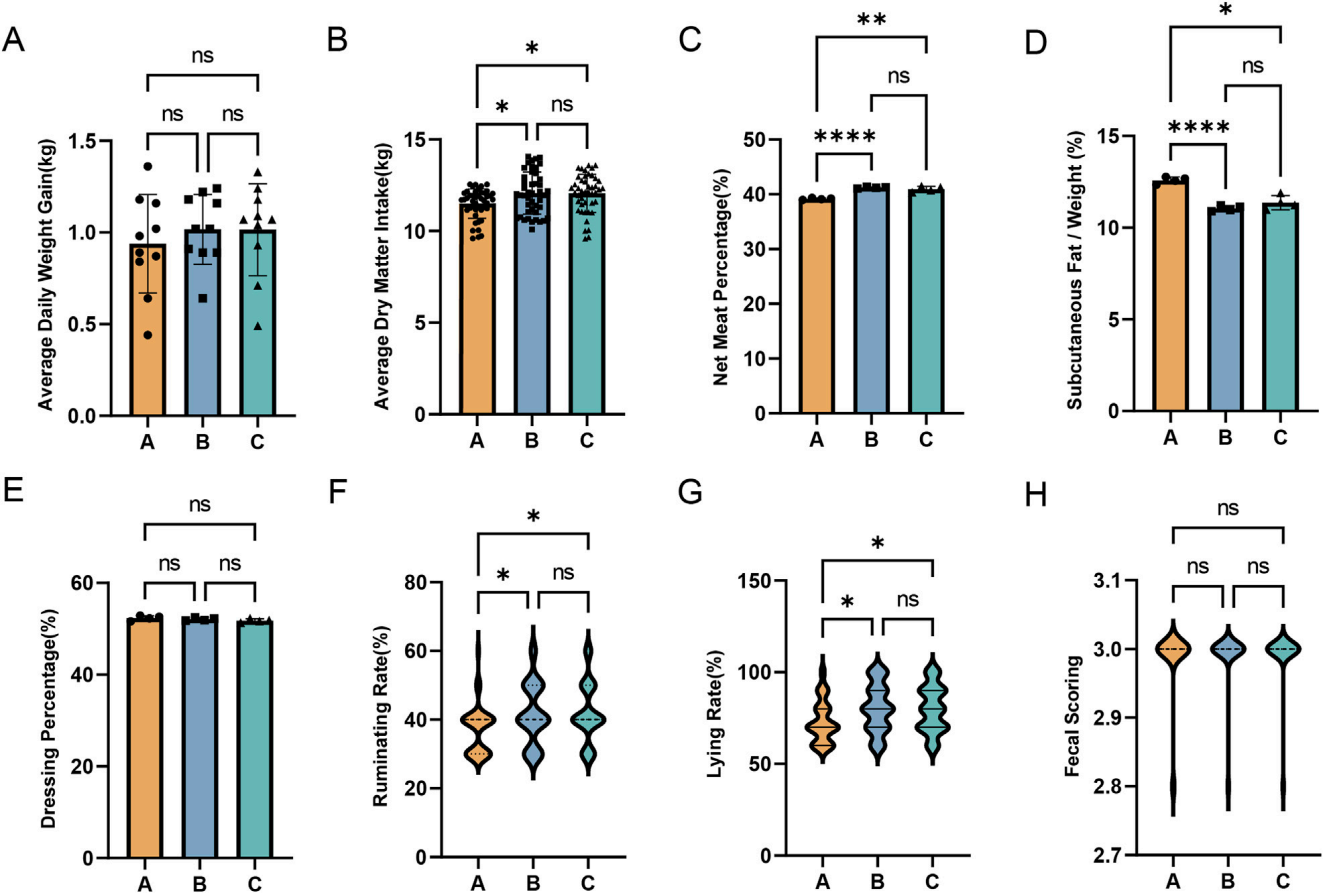
Effects of broccoli extract on the growth performance of castrated Holstein bulls. **(A)** Average daily weight gain of the three groups; **(B)** Average dry matter intake of the three groups; **(C)** Net meat percentage of the three groups; **(D)** The proportion of subcutaneous fat to weight in three groups.; **(E)** The dressing percentages of the three groups; **(F)** Ruminating rate of the three groups; **(G)** Lying rate of the three groups; **(H)** Fecal scoring of the three groups. A, control group; B, group fed 18 g broccoli extract for 45 days; C, group fed 15 g broccoli extract for 45 days.

To investigate the effects of broccoli extract additive on the growth performance of Holstein steers, we recorded various growth indicators at the beginning and end of the trial (Figure 2). The results showed no significant changes in body weight and fecal score in the experimental groups (*P > 0.05*). However, compared to the control group (Group A), Groups B and C showed significantly increased average dry matter intake, lying rate, and rumination rate (*P < 0.05*). Additionally, carcass data collected post-slaughter indicated that while there were no significant changes in dressing percentage in Groups B and C (*P* > 0.05), there was a significant increase in net meat percentage (*P < 0.01*) and a significant decrease in the proportion of subcutaneous fat in weight (*P < 0.05*).

### 3.2 Rumen microbiota diversity differences

**FIGURE 3:**
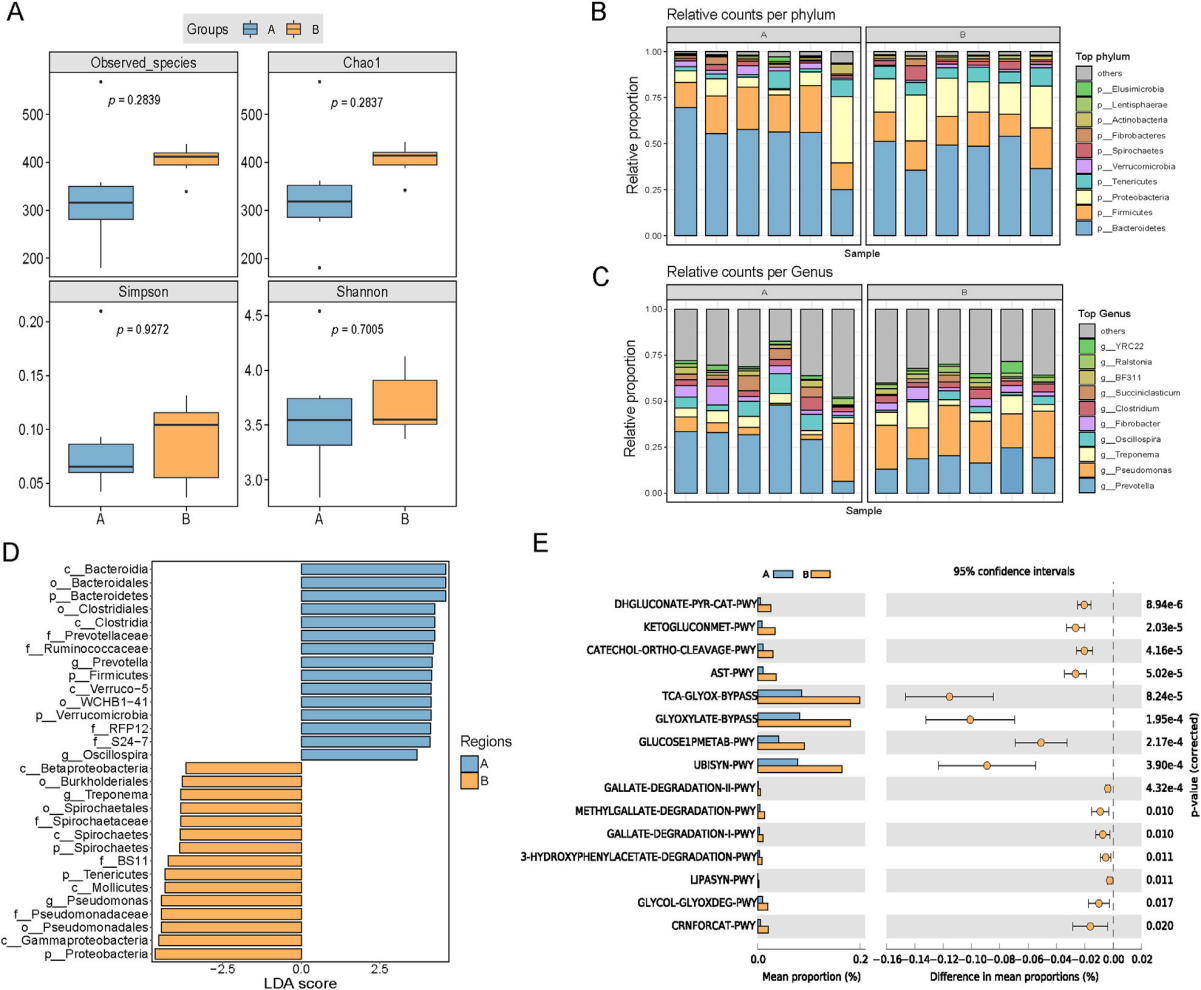
Effects of broccoli extract on rumen fluid microbial diversity of castrated Holstein bulls. **(A)** Influence of broccoli extract on the rumen fluid microbial α-diversity index. Group A: Control group; Group B: Treatment group supplemented with 18 g broccoli extract. **(B,C)** Impact of broccoli extract on the rumen fluid microbial community at the phylum **(B)** and genus **(C)** levels. **(D)** Comparison of the classification of rumen microbiota between two groups by linear discriminant analysis effect size (LefSe) method. **(E)** Functional prediction and differential metabolic pathways of rumen fluid microbiota. These pathways were inferred using PICRUSt2 and have yet to be experimentally validated.

Rumen function and metabolism play crucial roles in regulating feed intake, where the degradative capacity of the rumen may influence the performance of feed intake and rumination in animals (Terler et al., 2019). In the 16S rRNA analysis of rumen fluid, group B showed an increasing trend in the Simpson index, Chao1 index, and species coverage compared with those for group A, indicating that the rumen in group B had a more diverse microbial composition (Figure 3A). At the phylum level, the relative abundances of *Firmicutes* and *Bacteroidetes* decreased and those of *Proteobacteria* increased in group B compared with their relative abundances in group A (Figure 3B). *Prevotella* was the dominant genus in group A, whereas *Pseudomonas* was the dominant genus in group B (Figure 3C). Relative abundances of *Treponema*, *Pseudomonas*, and *Spirochaetales* in rumen fluid were significantly higher in group B compared with their abundances in group A (*P < 0.05*) (Figure 3D). We predicted potential metabolic pathways and functions within the microbial community based on a genus level analysis. The results showed that the rumen microbiota in group B may be associated with higher levels of glucose oxidation, ketoglutarate metabolism, L-arginine degradation, and pyruvate decarboxylation compared with their levels in the other two groups (Figure 3E). The above pathways were inferred via PICRUSt2 (Douglas et al., 2020) and remain to be experimentally validated in future research.

### 3.3 Non-targeted metabolomics of blood

**FIGURE 4:**
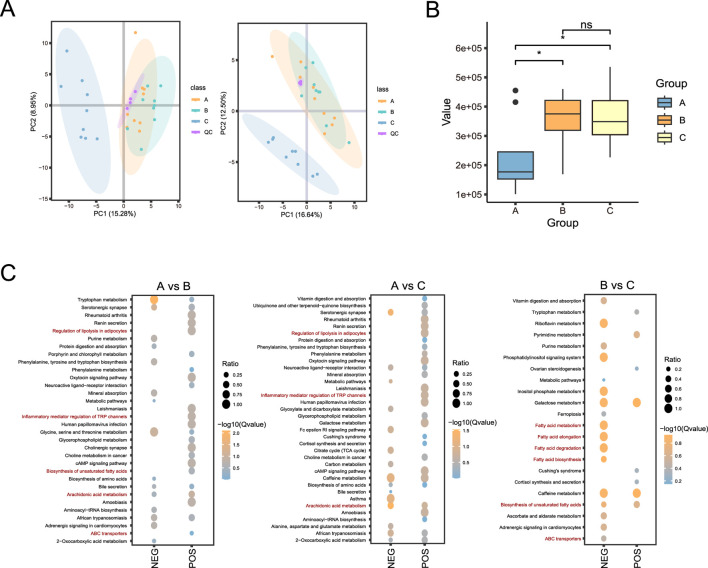
Non-targeted metabolomic analysis of blood of castrated Holstein bulls. **(A)** Principal component analysis (PCA) analysis of experimental samples and QC samples. **(B)** Box plot showing prostaglandin E2 levels in the three groups. **(C)** Bubble plots of the enriched KEGG pathways. A, control group; B, group fed 18 g broccoli extract for 45 days; C, group fed 15 g broccoli extract for 45 days; QC, quality control.

To gain insight into the effects of broccoli extract additive on the blood metabolism of castrated Holstein bulls, we conducted a comprehensive analysis of non-targeted metabolomics of their blood. Data assessment showed high repeatability and stability of the experiment, with a stable and reliable model (Figure 4A).

A total of 473 metabolites were identified in positive ion mode and 258 metabolites were identified in negative ion mode (Supplementary Table S1). Notably, the prostaglandin E2 levels were significantly higher in groups B and C groups, which were supplemented with broccoli extract, compared with its level in the control group (*P < 0.05*) (Figure 4B; Supplementary Table S1). The KEGG enrichment analysis (Figure 4C) indicated that in the positive and negative ions modes, differential metabolites between groups A and B were enriched mainly in the Arachidonic acid metabolism, Glycerophospholipid metabolism, and Tryptophan metabolism pathways. The differential metabolites in group A and group C were mainly enriched in Arachidonic acid metabolism, Galactose metabolism, Alanine, Aspartate and Glutamate metabolism pathways, while those in group B and group C were mainly enriched in unsaturated fatty acid biosynthesis and fatty acid degradation pathways. These results indicated that the differential metabolites were mainly enriched in pathways related to energy metabolism and lipid metabolism.

### 3.4 Transcriptomic differences

**FIGURE 5:**
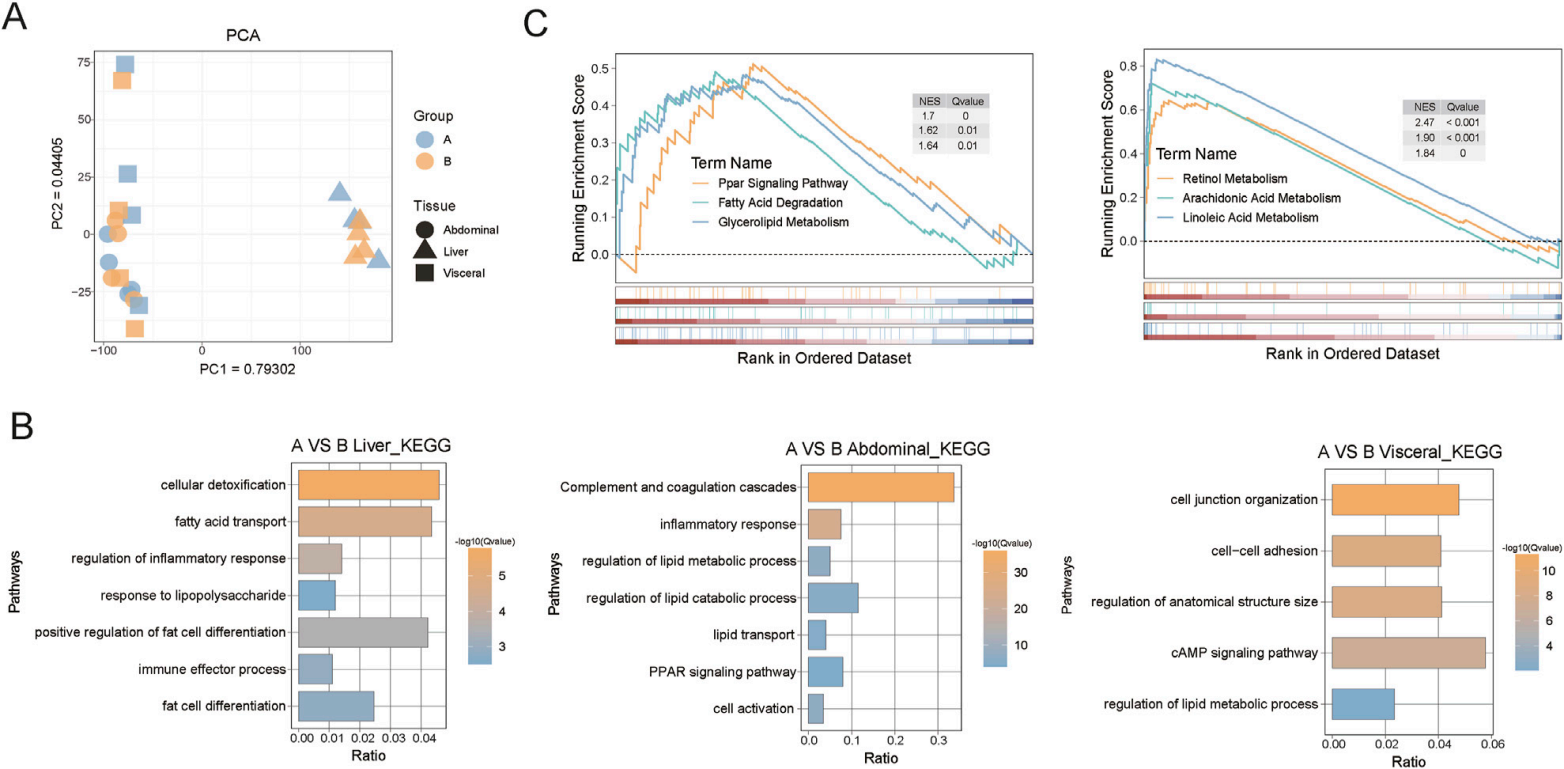
Transcriptome analysis of liver, abdominal fat, and visceral fat from castrated Holstein bulls. **(A)** Principal component analysis (PCA) plot of tissue samples; **(B)** Functional enrichment of differentially expressed genes in the three tissue types; **(C)** GSEA results for liver and abdominal fat. A, control group; B, group fed 18 g broccoli extract for 45 days.

To investigate the changes in the gene expression profiles of the liver and adipose tissues of castrated Holstein bulls after adding broccoli extract additives to their feed, we collected 24 samples of liver, abdominal fat, and visceral fat from groups A and B. The PCA plots show that the primary variance in the data was between the organs (Figure 5A).

The differentially expressed genes (DEGs) obtained by analysis of the transcriptome data of the three groups were annotated using the GO and KEGG databases. In group B, the liver tissue was enriched in cellular detoxification, fatty acid transport, and regulation of inflammatory response under the GO biological process category. In the adipose tissues of group B, the abdominal fat was enriched in inflammatory response, lipid metabolism, and decomposition processes, whereas the visceral fat was enriched in cell connection-related under the GO biological process category (Figure 5B). The GSEA showed that the broccoli extract had a significant regulatory effect on the metabolism of specific fatty acids in both the liver and adipose tissues (Figure 5C). The regulation of these genes may help to elucidate the potential mechanisms of the effects of broccoli extract on the metabolic health and growth of castrated Holstein bulls.

### 3.5 Integrated analysis of metabolomics and transcriptomics

**FIGURE 6:**
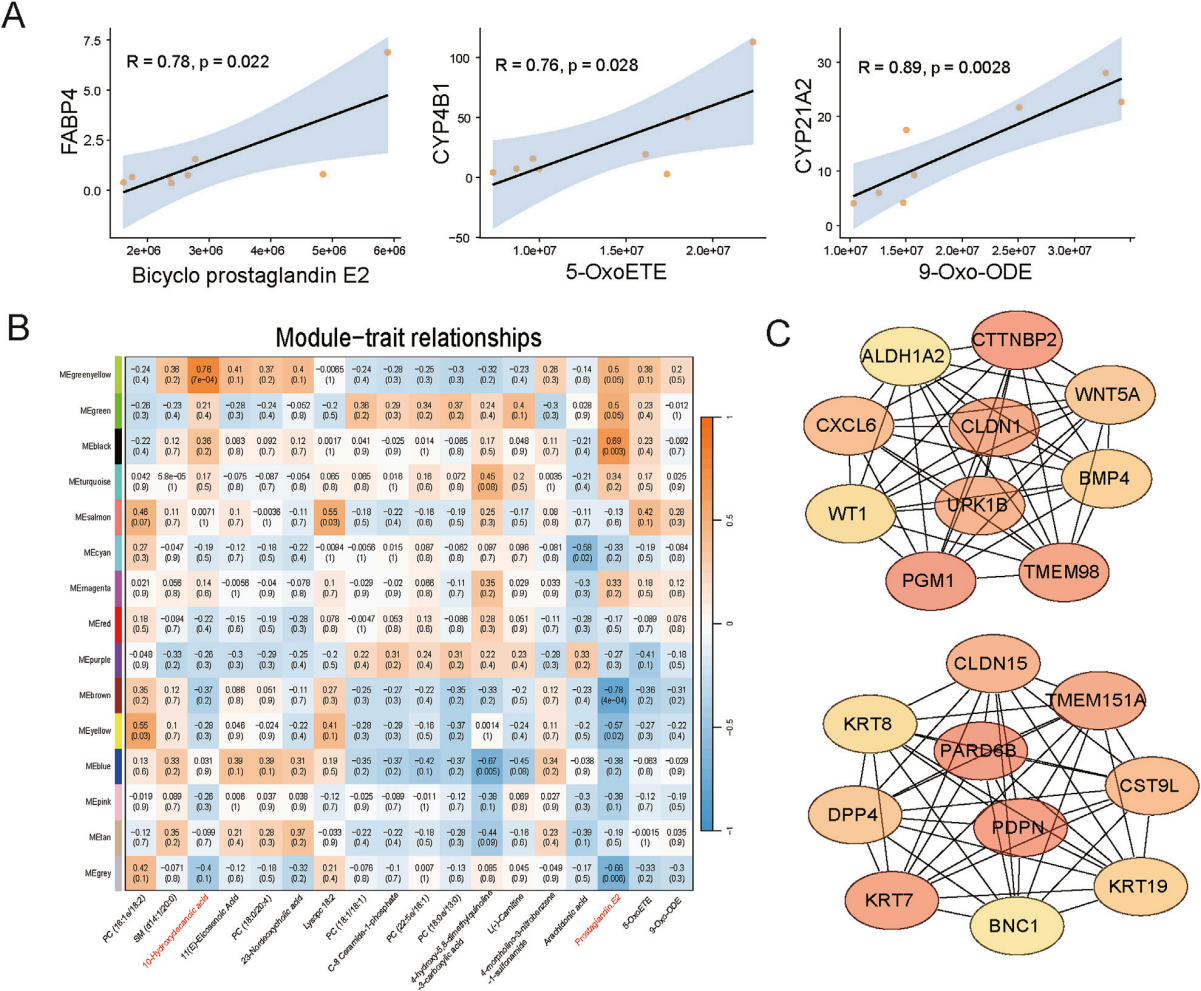
Integrated analysis of metabolomics and transcriptomics data. **(A)** Correlation fitting curve between differential metabolites and differentially expressed genes. **(B)** Heatmap showing the correlation between WGCNA gene modules and the abundance of differential metabolites. **(C)** Core genes identified by integrated WGCNA and Cytoscape analysis (Above, core genes of the black module; Below, core genes of the greenyellow module).

Correlation analysis between differential metabolites and DEGs indicated a strong correlation between lipid metabolism-related differential metabolites and some DEGs, including the fatty acid-binding protein gene (*FABP4*) and lipid transport protein gene (*ABCA9*) in liver, and CYP family genes (*CYP4B1*, *CYP1A1*, *CYP21A2*), and lipid-carrying protein genes (*APOC3*, *APOB*) in adipose tissue (Figure 6A; Supplementary Table S2). By calculating the correlation between WGCNA gene modules and the abundance of differential metabolites, we found that transcripts in the black module were highly correlated with prostaglandin E2, and transcripts in the greenyellow module were highly correlated with 10-hydroxydecanoic acid and 11(E)-eicosenoic acid (Figure 6B). Subsequently, we identified core genes in these two modules that may play important roles in the aforementioned associations (Figure 6C; Supplementary Table S3).

## 4 Discussion

We found significant differences in dry matter intake, lying time, and rumination rate of the castrated Holstein bulls that were fed different doses of broccoli extract, indicating the significant impact of broccoli extract supplementation on the productive performance and behavior of cattle. Notably, although we did not observe significant changes in the average daily gain of the cattle, we were surprised to find a significant increase in the net meat percentage and a significant decrease in the proportion of subcutaneous fat in body weight. This suggests several points: firstly, the fat deposition in the cattle gradually decreased and lipid metabolism progressively enhanced; secondly, the carcass traits of the cattle were effectively improved; thirdly, the feed utilization efficiency may have increased, resulting in more energy being used for lean meat synthesis rather than fat deposition. These results explain why there were no significant changes in body weight and clearly indicate the important role of the broccoli extract additive in bovine lipid metabolism.

The rumen function and metabolism play crucial roles in regulating feed intake, and the degradability activity of the rumen may affect the feed intake and performance of ruminant animals (Terler et al., 2019). The 16S rRNA results indicated that supplementation of broccoli extract in the diet increased the diversity of microbial communities in the rumen fluid of the bulls, and the genes of these microbial communities had significant functional differences in pathways such as Glucose oxidation, L-Arginine degradation, and Catechol degradation.

Additionally, Qin et al. (2010), Ley et al. (2008) demonstrated that the dominant microbial communities in the rumen of ruminants were *Firmicutes* and *Bacteroidales*, which is consistent with our results. However, the decrease in the relative abundance of *Bacteroidales* may be related to antagonism and competition with other microbial communities that increase in number and activity under the influence of plant extracts. Moreover, we observed an increased relative abundance of *Proteobacteria* in group B. Previous studies have indicated that members of this phylum are involved in cellulose degradation, suggesting that broccoli extract may enhance ruminal fiber digestion by promoting the growth of these fiber-degrading bacteria (Qiu et al., 2020). At the genus level, the relative abundance of *Pseudomonas* also appeared to be elevated in group B. *Pseudomonas* species have been reported to effectively degrade mycotoxins and secrete lipases that contribute to lipid breakdown under *in vitro* rumen conditions. Additionally, rumen-derived *Pseudomonas* strains are capable of degrading hydrocarbons and plant-derived fibers, thereby potentially facilitating the digestion of complex dietary components. Notably, a higher abundance of *Pseudomonas* in the rumen has been associated with improved feed digestibility in Hu sheep (Sun et al., 2022; Singh and Singh, 2022; Wang et al., 2022). Overall, these changes may contribute to improving the efficiency of feed utilization by cattle, thereby promoting increased feed intake. However, more research is needed to confirm how exactly these changes improve rumen health.

Consuming broccoli has been shown to contribute to lipid metabolism in animals and reduce cholesterol levels in the blood (Aranaz et al., 2020; Aborehab et al., 2016; Lee et al., 2009; Rodríguez-Cantú et al., 2011; Bahadoran et al., 2012), which is consistent with the enrichment pathways of the differential metabolites in our study. Notably, the prostaglandin E2 (PGE2) levels in the experimental groups significantly increased and were significantly enriched in the Arachidonic acid metabolism pathway, which is consistent with the findings of Okita et al. (2001). Prostaglandins are a class of twenty-carbon unsaturated fatty acids that are synthesized through the metabolism of arachidonic acid, and prostaglandin E2 (PGE2) is the most biologically active and extensively studied prostaglandin (Huang et al., 2023). Arachidonic acid is an important polyunsaturated fatty acid that is involved in regulating various biological processes and inflammatory responses (Lin and Yu, 2007; Tallima and El Ridi, 2018). Increased levels of arachidonic acid can produce bioactive substances such as prostaglandins, thromboxanes, and leukotrienes, which play important regulatory roles in processes such as inflammation, platelet aggregation, and vascular constriction. Arachidonic acid can also promote animal growth (Xu et al., 2022). Although there was no significant increase in average daily weight gain of the castrated Holstein bulls, the results indicate that adding broccoli extract had a positive effect on improving lipid metabolism and inflammation levels in the treated animals. It is noteworthy that, despite the absence of significant differences in growth performance between the 15 g and 18 g broccoli extract groups, clear distinctions in blood metabolic profiles were observed, indicating that these metabolic parameters may be more responsive to variations in extract dosage. Further studies employing more refined dosage gradients are warranted to elucidate the underlying dose-dependent effects.

Liver largely controls systemic nutrient and energy homeostasis, and abnormalities can lead to hepatic steatosis, steatohepatitis, steatofibrosis, and liver cancer. We found that gene expression in the liver indicated the role of broccoli extract in immune response and lipid metabolism Chen et al. (2016) also described the potential effects of dietary broccoli in counteracting the development of fatty liver and liver cancer in mice; the extract reduced hepatic triglyceride accumulation and induced effects similar to liver weight reduction. In addition, the PPAR signaling pathway was identified by GSEA, and peroxisome proliferator-activated receptors (PPARα, β/δ, and γ) are known to regulate lipid homeostasis. PPARα regulates lipid metabolism in the liver (Wang et al., 2020). Adipose tissue is an endocrine organ that releases adipokines, which are closely associated with lipid metabolism, homeostasis, and inflammation (Zhang et al., 2018). Maintaining lipid metabolism homeostasis is crucial for preventing and treating metabolic diseases such as diabetes and obesity (Hong et al., 2020). Supplementation of broccoli extract in rats for 10 weeks was shown to reduce their visceral fat mass and adipocyte size (Aranaz et al., 2019), which is consistent with our adipose tissue transcriptome results. At the gene expression level, we detected relatively weak regulation of the number and enrichment of genes in the bulls fed broccoli extract. The weak effect may be attributed to the short feeding duration being insufficient to induce significant fluctuations in the transcriptomes. Overall, the addition of broccoli extract to the feed had positive effects on lipid metabolism, inflammatory response, and immune-related pathways in the liver and adipose tissues, providing valuable evidence for improving the health and productivity of cattle.

The correlation analysis between transcriptomics and metabolomics showed strong correlation between differential metabolites in lipid metabolism and DEGs, which may reflect the important roles of these genes in regulating relevant metabolic pathways. By examining the highly correlated transcriptional modules in adipose tissue with the abundance of metabolites, especially lipid metabolism products, we identified core genes in the target module. Among them, the chemokine CXCL6 is closely associated with cell permeability, apoptosis, and proliferation (Zhou et al., 2023), and plays an important role in inflammation and immune responses (Zhou et al., 2023). Chemokines are known to selectively recruit and activate various cell types, causing inflammation in white adipose tissue by recruiting various immune cells and affecting glucose and fat metabolism in adipocytes (Liddle et al., 2017). DPP4 is an adipokine that plays a key role in obesity-induced inflammation and insulin resistance (Nauck et al., 2017; Zilleßen et al., 2016). Studies have shown that DPP4 levels and secretion in visceral fat are higher than those in subcutaneous fat (Schuetz et al., 2015). These results emphasize the tight coordination between gene expression levels and lipid metabolism products, implying that these genes may play crucial regulatory roles in specific metabolic pathways. However, the specific interactions between genes and metabolites require further experimental validation and analysis.

## 5 Conclusion

Broccoli extract significantly increased the feed intake, lying time, and rumination rate of castrated Holstein bulls, and effectively improved the composition of the rumen microbiome. Additionally, it significantly increased the net meat percentage of fattening cattle while reducing the proportion of subcutaneous fat in body weight, thereby improving carcass characteristics. Furthermore, it modulated inflammatory responses and promoted lipid metabolism at the metabolic and transcriptional levels. These effects may contribute to the growth and fattening of Holstein cattle in the future.

## Data Availability

The data presented in this study are deposited in the Genome Sequence Archive (GSA) under accession numbers CRA017433 (original raw 16S sequence data) and CRA017465 (original raw transcriptome sequencing data), accessible at https://ngdc.cncb.ac.cn/gsa. Additionally, the original metabolomic data are deposited in OMIX under accession number OMIX006776.
